# Microalgal therapeutic peptides: From biomass to bone tissue engineering

**DOI:** 10.1016/j.mtbio.2026.102938

**Published:** 2026-02-18

**Authors:** Diana Pacheco, Tatiana M.F. Patrício, Abílio J.F.N. Sobral, Telma Encarnação

**Affiliations:** a Coimbra Chemistry Centre-Institute of Molecular Sciences (CQC-IMS), Department of Chemistry, University of Coimbra, Coimbra, 3004-535, Portugal; b Centre for Rapid and Sustainable Product Development, Polytechnic of Leiria, Marinha Grande, 2430-028, Portugal; c SeaPower - Association for the Development of the Sea Economy, Industrial Park of Figueira da Foz, Rua das Acácias n°40 - A, Figueira da Foz, 3090-380, Portugal; d PTScience, Avenida do Atlântico, N 16, Office 5.07, Parque das Nações, Lisboa, 1990-019, Portugal

**Keywords:** Bioactive compounds, Biomimetic, Bone regeneration, Therapeutic peptides, Advanced delivery systems

## Abstract

Bone tissue engineering continues to face challenges in developing biomaterials that are both safe and biologically active, particularly in promoting integration with native tissue. Traditional synthetic materials often lack cellular compatibility, driving research toward natural and biomimetic alternatives. In this context, microalgae have a diverse metabolic profile, producing several biologically active compounds (i.e. lipids, carbohydrates, pigments) with therapeutic potential for bone regeneration. Among these, peptides gain relevance due to their high cellular compatibility, osteogenic activity and tunable properties. Herein, this review provides a comprehensive and critical overview of microalgae-derived peptides, covering their manufacturing process. It covers the entire workflow from protein extraction to peptide purification and characterization. It summarizes their biological properties and therapeutic applications in bone regeneration and examines their status in clinical studies alongside the main regulatory and translational challenges. Particular focus will be given to the combination of advanced delivery systems for using microalgae therapeutic peptides to develop patient-specific implants. Overall, this review emphasizes the significance of microalgae as a versatile and sustainable resource to extract therapeutic peptides and to develop the next generation of biomaterials in bone regenerative medicine.

## Highlights

• Microalgae are a sustainable source of peptides with biological activity.
• Advances in extraction and characterization techniques enhance peptide quality and reproducibility.
• Microalgal peptide-based hydrogels support 3D printing of patient-specific bone implants.
• Scalable production and regulatory considerations are essential for clinical translation.
• Microalgal peptides offer a promising platform for next-generation biomaterials in regenerative medicine.

## Graphical abstract

**Figure:**
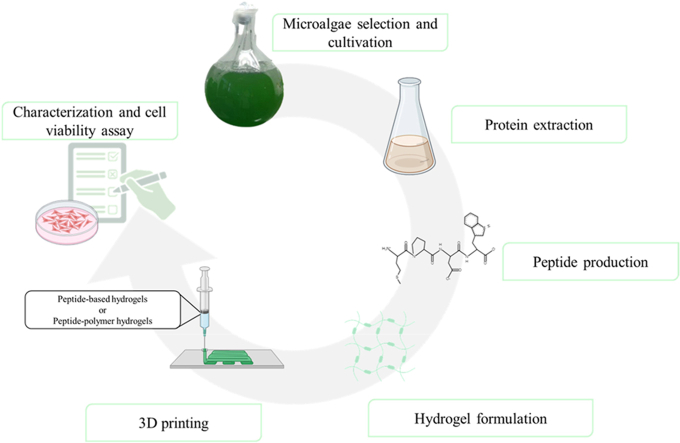

## 1 Introduction

The global increase in life expectancy and the prevalence of bone diseases such as osteoporosis and osteosarcoma have amplified the occurrence of bone defects [1]. While bone has a natural capacity for regeneration, critical-size fractures (>2.5 cm) often require surgical intervention [2]. Current Food and Drug Administration (FDA) approved treatments, including autografts, allografts, xenografts, and synthetic implants, face limitations [3]. Current clinical approaches, such as autografts are considered the gold standard due to their high osteoconductivity. However, their application is constrained by limited availability and donor-site complications. Allografts and xenografts, though accessible, carry risks such as infection, poor vascularization, and ethical concerns, while synthetic implants often lack bioactivity [4]. These challenges have redirected research towards biomimetic and naturally derived materials that mimic the hierarchical composition and regenerative cues of the native bone [3]. To overcome these limitations, increasing attention has been directed toward small therapeutic peptides. In comparison with proteins, peptides offer improved control over biological activity and better control of their release.

Compared to clinically used osteogenic agents, such as bone morphogenetic proteins (BMPs) and platelet-rich plasma (PRP), therapeutic peptides offer a distinct balance between bioactivity, safety and cost-effectiveness. BMPs exhibits strong osteoinductive potential [[5], [6], [7]], its clinical use is limited by high cost, burst release, and adverse inflammatory responses [8,9]. While PRP suffers from donor variability and inconsistent therapeutic outcomes [10]. In contrast, therapeutic peptides demonstrate moderate but multi-target osteogenic activity, combined with low immunogenicity and high production scalability. Unlike synthetic peptides, which require complex chemical synthesis, biologically derived therapeutic peptides can be produced sustainably with tunable composition and favorable integration into biomaterial-based delivery systems. Despite these advantages, most therapeutic peptides are still obtained through cost-intensive synthetic or recombinant technologies [11]. Consequently, there is a growing interest in identifying natural and sustainable peptide sources that maintain biological activity and compatibility [12]. Synthesized peptides are commonly used for scaffold biofunctionalization, such as RGD, FHRRIKA, PSHRN and YGFGG [12]. Recently, naturally derived materials have gained increasing attention as alternative agents for biofunctionalization. Their application presents advantages such as reduced production costs, lower cytotoxicity and enhanced cellular compatibility [13,14].

In this context, microalgae are recognized as a sustainable source of therapeutic peptides, due to their high protein content and fast growth rate [15]. Some studies have reported promising osteogenic effects of peptides derived from microalgae. For instance, a tetrameric peptide with osteogenic activity was isolated from bio-based hydrolysates byproducts from *Nannochloropsis oculata* [16]. This peptide promoted osteoblast differentiation and increased the expression of osteoblast phenotypic indicators like alkaline phosphatase activity, osteocalcin, collagen type I, and bone mineralization in murine mesenchymal stem cells [16]. Additional evidence from a study using a mix of peptides from *Arthrospira platensis* reported increased expression of RUNX2, osteocalcin, and β-catenin, along with higher ALP activity in human amniotic mesenchymal stem cells [17].

Although Arrieta Payares et al. (2023) [12] have addressed the role of microalgae molecules on bone regeneration processes, a comprehensive synthesis focusing on microalgae-derived peptides is still lacking, especially regarding the relationship between their production strategies and potential applications in bone tissue engineering. Therefore, this review critically analyzes the production, extraction and characterization of microalgal peptides, with an emphasis on their osteogenic mechanisms and translational feasibility. In addition, it emphasizes advanced delivery systems approaches of microalgae therapeutic peptides for bone tissue engineering.

This review considered peer-reviewed articles published in English between 2010 and 2026. The literature search focused on studies investigating the therapeutic activities of peptides derived from microalgae, including anti-inflammatory, antioxidant, antibacterial and/or osteogenic. Eligible publications included *in vitro*, *in vivo*, and clinical studies. Reviews, research articles, and relevant mechanistic studies were considered when they contributed to the understanding of peptide bioactivity or signaling pathways. The literature search was conducted using the following databases: Web of Science, Scopus, PubMed, and B-On. Patent related information was retrieved from Espacenet and Google Patents. Clinical trial data was identified using NCBI databases. To address regulatory considerations, publicly available regulatory guidance from European Medicines Agency (EMA) was reviewed. Search terms were used individually and in combination, and keywords, such as microalgae, peptides, bone regeneration, hydrogels, osteogenesis, tissue engineering and additive manufacturing.

## 2 Microalgae as a potential source of therapeutic peptides

Microalgae have emerged as a renewable source of biologically active molecules which are ‘‘Generally Regarded As Safe’’ (GRAS) for food and feed applications by FDA [18]. While this designation supports nutritional use, it also enhances their potential for biomedical translation, as GRAS status implies prior evidence of non-toxicity and biological compatibility.

Among the major classes of macromolecules produced by microalgae (i.e. lipids, carbohydrates), proteins represent the most abundant fraction and the primary source of peptides. Microalgae genus such as, *Spirulina* and *Chlorella* contain 50–70% and 50–60% protein by dry weight, respectively, while *Porphyridium cruentum* and *Nannochloropsis* species range between 28 and 50% protein dry weight [[19], [20], [21], [22]]. In contrast, conventional plant protein sources (i.e. soybeans, almonds, walnuts, quinoa) and animal-derived proteins (i.e. beef, pork, chicken, and eggs) generally provide 12–56% and 20–31% dry weight, respectively [23]. Combined with fast growth rates and scalable cultivation systems, this high protein yield makes microalgae particularly attractive feedstocks for the sustainable production of therapeutic peptides.

While protein abundance is an important pre-requisite, the therapeutic potential depends on the peptides amino acid sequence of the hydrolyzed proteins. As a result, the amino acid content of microalgae proteins determines the variety of peptides formed during hydrolysis, and thus their bone tissue engineering potential. Amino acids including serine, proline, aspartic acid, and glutamic acid, which are found in both collagenous and non-collagenous bone matrix proteins, are critical for mineralization processes [24,25]. While essential amino acids, such as lysine, threonine, methionine, tryptophan, and isoleucine may promote osteoblast proliferation and differentiation [26]. Hence, proteins enriched in these amino acids are more likely to yield peptides with osteogenic and mineralization promoting activity.

Commercially available species, such as *Chlorella* sp., *Nannochloropsis* sp., and *Arthrospira* sp., are highlighted for their total amino acid (TAA) diversity and high amount when compared with a typical protein source (Table 1). Previous research suggests that TAA profiles are not species-specific, as no consistent species or phylum specific patterns have been observed. Instead, protein concentration is strongly influenced by cultivation conditions, which in turn affects the composition and functionality of the peptides generated following hydrolysis. To increase microalgae protein content and quality during microalgae production, optimal environmental parameters, including temperature, light intensity, salinity, and pH, as reviewed by Zheng et al. (2025) [30]. Optimal cultivation parameters rely not only on the microalgal species, but also on whether an outdoor or indoor growing system is used, and the growth methods employed. As a result of their compositional diversity, tunability and sustainability, microalgae-derived peptides are emerging as a next-generation biomolecular cues for bone repair [31].

**Table 1:** Total amino acid profile of selected microalgae (g 100g protein^−1^) compared to egg albumin.

Amino acid	*Chlorella* sp. (Chlorophyta)	*Nannochloropsis* sp. (Ochrophyta)	*Arthrospira* sp. (Cyanobacteria)	Egg albumin
**Essential amino acid (g 100g protein^−1^)**				
Arginine	6.4	7.4	4.4	6.2
Histidine	2	2.3	1	2.4
Isoleucine	3.8	5.6	3.6	6.6
Leucine	7.8	11	5.5	8.8
Lysine	8.4	8.5	3	5.3
Methionine	2.2	3.5	1.4	5
Phenylalanine	5	6.2	2.8	5.8
Threonine	4.8	5.4	3.3	5
Tryptophan	2.1	2.8	1	1.7
Valine	5.5	7.1	4.5	5.3
**Non-Essential amino acid (g 100g protein^−1^)**				
Alanine	7.9	7.1	4.7	-
Aspartic acid	9	11.4	6	11
Cysteine	1.4	1.6	0.7	2.3
Glutamic acid	11.6	14.1	9.2	12.6
Glycine	5.8	7.5	3.2	4.2
Proline	4.8	11.2	2.7	4.2
Serine	4.1	5.6	3.3	6.9
Tyrosine	3.4	4.2	3	4.2
Reference	[27]	[28]	[29]	[27]

## 3 Microalgae therapeutic peptide production

**Fig. 1:**
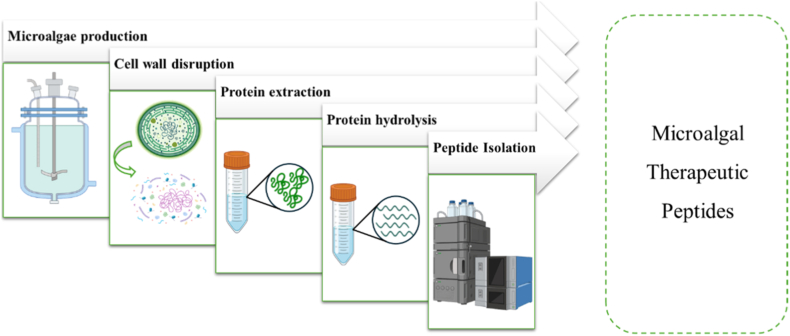
Workflow process to produce microalgal therapeutic peptides.

Microalgae therapeutic peptides are produced through a series of processes that includes microalgae production, cell disruption, protein extraction, protein hydrolysis, and peptide isolation and characterization (Fig. 1). Each process influences peptide yield, purity, and preservation of peptide therapeutic effect for bone regeneration. Recent advances in mild extraction strategies, green solvents and high-resolution analytical tools have improved the recovery and identification of microalgae therapeutic peptides with bone relevant biological activity.

### 3.1 Protein extraction

Protein extraction begins with controlled microalgae cultivation, which directly influences protein yield [32]. Optimization of microalgae growth parameters (i.e. light, nutrients) has been shown to modulate protein yield, making cultivation an important step of peptide design [33]. Protein yield is strongly dependent on species-specific cell wall structural and biochemical composition, as the chloroplast thylakoid membrane contain the major protein fraction [34]. Consequently, the selection of cell disruption methods not only affect the extraction yield (Table 2), but also peptide integrity and therapeutic activity.

**Table 2:** Effect of microalgal biomass cell disruption method on protein extraction. NR – Non reported.

Type of cell disruption method	Species	Conditions	Yield of protein extraction (%)	Advantages	Disadvantages	Reference
Pulsed electric Field	*C. vulgaris*	45 °C at 17.1 kV/cm for 5μs	4.4	• Non-thermal cell permeabilization method • Allows the extraction of small molecular weight proteins • Selective method • Low operational costs	• Could be used as a supplementary treatment, but it is not an efficient disruption method for complete protein extraction.	[[35], [36], [37]]
	*Chlorella* sp.	4.5 kV/cm for 15 pulses for 22 ms, in water	<10			
	*H. pluvialis*	3 kV/cm for 2 ms	10			
	*N. salina*	6 kV/cm, 2 pulses for 2 ms	>10			
Sonication	*Nannochloropsis* sp.	20 kHz, 100% amplitude, 4 min, 300 W	20-50	• Sonication of microalgae cells permeabilizes both the cell wall and the membrane • Releases cytoplasmatic proteins • Low operational costs • Easily scalable	• High field strength is needed when small cell size • High voltages, sonication induces non-homogeneous cell disruption • Sonication alone is not sufficient for complete extraction of proteins • Expensive equipment • Heat production	[[38], [39], [40], [41]].
	*A. platensis*	37 Hz, 30 °C, 35 min followed by 50 min agitation	75			
	Algal mix Chlorococcale, Chlorophyceae class	30 kHz, 50W	60			

Bead milling	*C. vulgaris*	500 s, zirconia beads	87.5	• High efficiency • High loading capacity • Good temperature control • Easily scalable • Equipment commercially available	• High energy demand • Non-selective procedure • Formation of cell debris	[[41], [42], [43], [44], [45]]
	*Chlorella* sp.	90 min, 0.6–0.8 mm zirconia beads, 85% filling	NR			
	*C. vulgaris*	40 min, 1–1.6 mm zirconium silicate beads at 2500 rpm	96			
	*N. gaditana*	20min, 0.5 mm beads, zirconium, 65% filling	>90			
High-pressure homogenization	*C. vulgaris* *H. pluvialis* *A. platensis*	2 passes (2700 bar)	53 41 78	• High efficiency • Does not require cell drying • Suitable for large volumes processing	• High energy consumption • Hard to scale up	[43,[45], [46], [47]]
	*N. gaditana*	1 pass (1000 bar)	49			
	*C. vulgaris*	3 passes (1000 bar)	75			
	*Nannochloropsis* sp.	6 passes (1500 bar)	91			
Enzymatic	*N. gaditana*	Alcalase, 5h, 5% dry matter, pH 8.5	35	• Environmentally friendly • Non-toxic • Low energy requirements	• Expensive • Normally requires a cell permeabilization or disruption step	[43,[48], [49], [50]]
	*C. reinhardtii*	Autolysin, 5 h, pH 7.5, 37 °C	50			
	*C. ellipsoidea* *C. vulgaris*	4% onozuca, 2% macerozyme, and 1% pectinase, pH 6, 25 °C	NR			
Acid/Base	*A. platensis* *N. oceanica*	3 M hydrochloric acid in a water bath at 35 °C and 100 rpm for 15 min	NR	• Simple • Enables continuous operation • Scalable	• Environmental concerns • Chemical contamination	[51,52]

Mechanical and physical procedures, such as bead milling, sonication, or high-pressure homogenization, typically produce higher protein yields, often exceeding 70 to 90% in species like *Chlorella vulgaris* [42], *Arthrospira platensis* [38], and *Nannochloropsis* sp. [43]. However, these methods can cause localized heating, and non-uniform cell disruption, increasing the risk of protein denaturation, which may compromise the therapeutic potential of the isolated peptides. Pulsed electric fields offer a non-thermal option but often result in reduced extraction yields (<10%) [35,36].

Enzymatic lysis, on the other hand, provides a bioactivity-preserving alternative by operating under mild conditions that better maintain protein conformation and peptide integrity, although at lower yields. While it is an environmentally friendly technique that retains protein structure and bioactivity with yields of around 50% [43,48,49], it is limited by high cost and long processing times.

Acid and base treatments are simple and scalable, but they pose environmental and health hazards due to the chemicals that could remain [51,52]. Additionally, acid and base treatments may induce peptide denaturation or side-chain modifications (e.g., deamidation of asparagine residues under acidic conditions), thereby compromising their osteoinductive activity.

Therefore, selecting a disruptive technique needs balancing yield, scalability, and preservation of the peptide therapeutic activity. Overall, for bone tissue engineering applications, enzymatic or hybrid extraction strategies (e.g. mild mechanical processes, followed by enzymatic treatment) represent the most suitable compromise between yield, scalability, and preservation of peptide therapeutic activity.

### 3.2 Protein hydrolysis: controlling peptide functionality

Following extraction, proteins must be hydrolyzed to produce therapeutic peptides. Chemical hydrolysis typically employs strong acids or bases to cleave peptide bonds. However, it frequently compromises peptide integrity, due to a variety of side reactions, including oxidation, deamidation, isomerization or β-elimination [53]. For instance, amino acid asparagine is pH sensitive. Under acidic conditions, deamidation occurs, resulting in the formation of aspartic acid. While peptides containing asparagine residues are more susceptible to isomerization and subsequent hydrolysis. Whereas aromatic amino acids such as histidine, tyrosine, tryptophan, phenylalanine, and sulphur-containing amino acids such as methionine and cysteine are more susceptible to oxidation. Whereas β-elimination predominantly affects peptides bearing an electron-withdrawing substituent located on the side chain Cβ position involving removal of HS- or HO- from cysteine, serine, and threonine, especially when exposed to alkaline pH and high temperatures [53]. Overall, due to these undesirable side reactions, chemical hydrolysis is often considered unsuitable to obtain therapeutic peptides because reproducibility and functional or structural preservation is required.

In contrast, enzymatic hydrolysis has become the preferred strategy due to its substrate specificity, mild reaction conditions, and lower risk of toxic contamination. Additionally, enzymatic methods improve control over peptide length and sequence, which are essential for guaranteeing therapeutic activity, and improving reproducibility. Moreover, recent developments *in silico* enzymatic hydrolysis provide useful tools for predicting peptide release and activity. Tools, such as PeptideCutter, operate on the principle of primary sequence mapping, using a library of known proteolytic specificities to simulate enzyme-specific cleavage sites. While databases, such as BIOPEP-UWM uses fragment-matching algorithms to cross-reference sequences against experimentally validated bioactive motifs [54]. In the case of novel peptides, Peptide Ranker employs N-to-1 neural networks to predict a peptide's probability of being bioactive based on its amino acid composition and positioning [55].

Overall, the selection of the hydrolysis method will influence the molecular weight distribution of the resulting peptides. Harsh hydrolysis may lead to over-fragmentation or chemical modification, resulting in the loss of the peptides’ therapeutic activity. While mild enzymatic hydrolysis or hybrid hydrolysis techniques may preserve their functional properties [56,57].

### 3.3 Peptides isolation and characterization

The extraction and characterization of therapeutic peptides is critical to ensure their stability, consistency, and therapeutic efficacy. Considering vulnerability to degradation, improved and standardized isolation and characterization approaches are required. Hence, advances in separation and analytical technologies have assisted the development of this field of research [58]. The overall goal of peptides characterization is to confirm the molecular identity, evaluate the purity and identify potential toxic byproducts, following the guidelines established by the International Conference on Harmonization (ICH) guidelines [59].

Chromatographic and membrane-based methods remain the most used technologies for peptide purification [60]. Among these, Reverse Phase High Performance Liquid Chromatography (RP-HPLC) is the most frequently used method for peptide analysis due to its high resolution, recovery yield and reproducibility. As a result, 60% of the therapeutic peptides in pharmacopeia compendia were identified using RP-HPLC. Other methods have been used as Size Exclusion Chromatography (SEC), Hydrophilic Interaction Liquid Chromatography, Ion Exchange Liquid Chromatography, and Capillary electrophoresis (CE) are separation technologies that are either used in addition to, or in combination with RP-HPLC. SEC and ion exchange chromatography are often used to assess peptide size distribution and charge heterogeneity. While the other indicated separation techniques can be used for peptide analysis to determine their identification, quantity, purity, and stability [58].

For large scale fractioning, membrane-based techniques, such as microfiltration, ultrafiltration, nanofiltration, reverse osmosis, dialysis, and electro-membrane filtration offer a cost-effective and scalable alternative to chromatography [60]. However, their lower selectivity limits their use to peptide concentration and size-based pre-isolation rather than precise purification. Electro-membrane filtration, which combines charge interaction and size exclusion, offers a promising alternative by improving selectivity, enhancing peptide migration rates, and reducing membrane fouling [61].

Mass spectrometry (MS) techniques have become the primary analytical tool for identifying amino acid sequences and post-translational modifications in therapeutic peptides. Additionally, it can be employed to deduce its sequence and it's commonly used in combination with techniques like Liquid Chromatography (LC), Light scattering, Electrospray Ionization (ESI), and Matrix-Assisted Laser Desorption/Ionization (MALDI) [62]. Due to its high sensitivity, small sample requirement and potential for high-throughput analysis, MS has gained relevance in pharmaceutical peptides analysis. In addition, coupling liquid chromatography to MS has enhanced the field of novel pharmaceuticals development. MS works by measuring the mass-to-charge ratio (*m*/*z*) of gas-phase ions.

This advancement is a direct result of the introduction of soft ionization methods such as electrospray ionization and matrix-assisted laser desorption/ionization (MALDI) [63,64]. These ionization approaches have enabled the ionization of biomolecules and their transfer to the gas phase without dissociation. Electrospray and MALDI are both suitable ionization techniques, and when combined with a tandem mass spectrometer, they provide useful analytical instruments for biological study. MALDI Q-TOF, MALDI TOF-TOF, and a new-generation Q-TOF with MALDI and nano-LC choices, all of which have significant potential for peptide analysis [65]. In addition, coupling MS with *de novo* sequencing is an effective technique for determining a peptide amino acid sequence directly from tandem MS. The advantage of this technique is that it allows the identification of novel peptides that are not present in existing databases [66]. In complementarity, classical methods such as Edman degradation remain relevant to determine N- or C- terminal sequences of small therapeutic peptides [67].

Nuclear Magnetic Resonance (NMR) is a technique used to analyze the structure of macromolecules at atomic resolution. This technology can be used to obtain additional structural information by measuring chemical shifts, torsion angle restraints from scalar coupling measurements, and distance restraints from nuclear Overhauser effect experiments of nuclei probes (^1^H, ^13^C, ^15^N) that are scattered throughout the backbone and side chains of peptides [68]. Additionally, vibrational spectroscopy is widely used to study the secondary structures of peptides and proteins. Peptides have amide bands that are sensitive to the structure of the peptide backbone in infrared and Raman spectroscopy, with changes in secondary structure resulting in variances in the amide peaks [69,70].

Overall, the combination of advanced extraction, purification, and analytical techniques provides the basis for scalable and reproducible production of microalgae therapeutic peptides. Continuous improvement of these procedures, particularly through sustainable and cost-effective techniques, will be required to move microalgae therapeutic peptides from research to clinical-grade biomaterials.

## 4 Role of microalgae therapeutic peptide in modulating bone microenvironment

**Fig. 2:**
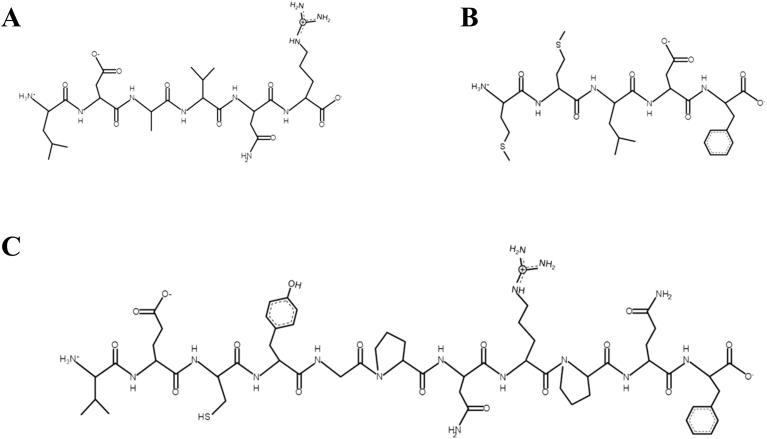
Chemical structure of microalgae therapeutic peptides with anti-inflammatory activity: **A –** LDAVNR, **B –** MMLDF, and **C –** VECYGPNRPQF. Peptide structures were generated using PepDraw [77].

Microalgae therapeutic peptides have emerged as potential modulators of cellular processes involved in bone tissue regeneration [71]. These include osteogenic, antioxidant, anti-inflammatory, and antibacterial characteristics, all of which promote bone tissue regeneration (Table 3). Bone regeneration involves a coordinated series of biological processes, including osteoimmunomodulation, neuroregulation, angiogenesis and bone formation [76]. In this first process, inflammation is essential for bone healing. However, excessive or prolonged inflammatory response can impair bone formation. The early stage of bone injury is characterized by a high concentration of pro-inflammatory macrophages (M1) and cytokine production, which, if unresolved, suppress osteoblast function and promote bone resorption. Therefore, controlling inflammation in a stage-dependent manner is a critical aspect for effective bone regeneration. Microalgae therapeutic peptides exhibit anti-inflammatory properties that may support bone healing by modulating the inflammatory microenvironment. For instance, peptides isolated from *Arthrospira maxima*, LDAVNR (Fig. 2A) and MMLDF (Fig. 2B), reduced histamine release and interleukin-8 (IL-8) in rat basophilic leukemia cells (RBL-2H3) and human umbilical vein endothelial cells (EA.hy926) [1]. Therefore, indicating attenuation of early inflammatory signaling. Similarly, the peptide VECYGPNRPQF (Fig. 2C) derived from *Chlorella pyrenoidosa* inhibited pro-inflammatory cytokines. Specifically, it reduced tumor necrosis factor α (TNF- α) and interleukin-6 (IL-6) in macrophages. Its effects were further evaluated in endothelial cells (SVEC4-10) to assess attenuation of inflammation-induced endothelial activation. By limiting inflammatory mediator release, these peptides may indirectly favor osteogenic differentiation and bone formation. Although *in vivo* evidence is lacking, these findings highlight the potential of microalgae therapeutic peptides for designing biomaterials that control inflammation during the early stages of bone regeneration.

**Table 3:** Microalgal therapeutic peptides with bone tissue engineering potential. Non-reported: NR.

Species	Hydrolytic Method	Peptide Sequence	Source	Peptide Concentration	Bioactivity	Primary Outcomes	Ref.
*Arthrospira maxima*	Trypsin alfa-chymotrypsin pepsin	LDAVNR	Extracted	200 μM	Anti-inflammatory	• Inhibits histamine production and release in mast cells • Reduces IL-8 in endhotelial cells • Suppression of intracellular ROS	(1)
		MMLDF	Extracted	200 μM	Anti-inflammatory		
*Chlorella pyrenoidosa*	Pepsin, flavourzyme, alcalase, and papain	VECYGPNRPQF	Extracted	9-38 μM	Anti-inflammatory	• Inhibitor of TNF-α and IL-6 production in macrophages	(72)
*Limnospira maxima*	Pepsin	KLENCNYAVELGK	Extracted	NR	Antibacterial	• Antibacterial activity against *E. coli* and *S. aureus*	(73)

*Arthrospira platensis*	NR	GGTCVIRGCVPKKLM	Identified/Chemically synthesized	25 μM	Antioxidant	• Scavenges superoxide and hydroxyl radicals • Modulates intracellular oxidative stress	(74)
*Chlorella sorokiniana*	Thermolysin	LSSATSAPS	Extracted	NR	Antioxidant	• C80% fraction exhibited the strongest antioxidant activity	(75)
		AGLYGHPQTQEE	Extracted	NR	Antioxidant		
*Nannochloropsis oculata*	Alcalase	MPDW	Extracted	20-500 μg/mL	Osteoblast differentiation	• Promotes osteoblast differentiation in MG-63 and D1 cells • Enhances bone marker expression (ALP, osteocalcin, collagen I)	(16)
*Arthrospira platensis*	Trypsin endonuclease	NR	Extracted	100-700 μg/mL	Osteogenic differentiation	• 3 kDa peptide fractions exhibited positive impact on osteogenic differentiation in hAMSCs • High expression of Runx2, osteocalcin and β-catenin • Increased ALP the activity and calcium deposition in the extracellular matrix	(17)

**Fig. 3:**
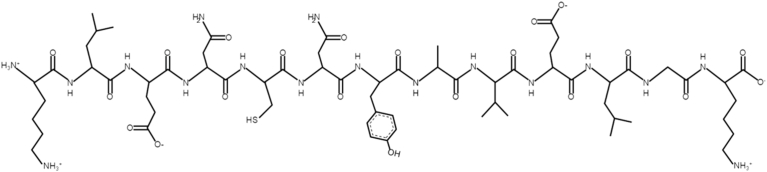
Chemical structure of microalgae therapeutic peptides with antibacterial activity. Peptide structures were generated using PepDraw [77].

Bacterial infections related to bone fractures are a major complication in orthopedic surgeries. They often cause delayed bone healing, prolonged hospitalization, and a negative impact on the patient's life quality [78]. Among the pathogens associated with bacterial infections related to bone fractures, *Staphylococcus aureus* is the most frequently reported [78]. Hence, it highlights the need for biomaterials with targeted antibacterial activity [79]. Bacterial cell membranes are negatively charged, while many peptides are positively charged. This electrostatic interaction leads to bacterial cell membrane disruption [80]. In this context, the microalgae therapeutic peptide extracted from *Limnospira maxima* (KLENCNYAVELGK, Fig. 3) demonstrated to be effective against both *Escherichia coli* and *Staphylococcus aureus*, suggesting its potential use in infection-responsive biomaterial design [73].

**Fig. 4:**
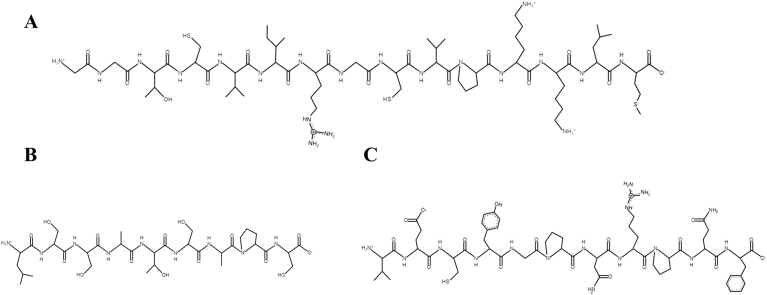
Chemical structure of microalgae therapeutic peptides with antioxidant activity: **A –** GGTCVIRGCVPKKLM, **B –** LSSATSAPS, and **C –** AGLYGHPQTQEE. Peptide structures were generated using PepDraw [77].

High levels of reactive oxygen species (ROS) at bone injury sites impair osteoblast differentiation and promote osteoclast activity, thereby delaying bone regeneration. In this context, the antioxidant bioactivity of microalgal therapeutic peptides can also contribute in decreasing oxidative stress at the injury site, enhancing the microenvironment for bone healing [74,75]. For example, the peptide GGTCVIRGCVPKKLM (Fig. 4A), identified from *Arthrospira platensis* and chemically synthesized, demonstrated high antioxidant activity by scavenging superoxide and hydroxyl radicals and modulating intracellular oxidative stress [74]. In another study, the peptides LSSATSAPS (Fig. 4B) and AGLYGHPQTQEE (Fig. 4C), isolated from *Chlorella sorokiniana*, following thermolysin hydrolysis, were identified in the C80% fraction of the hydrolysate, which exhibited the highest DPPH inhibition (22.04%). Such antioxidant effects may contribute to maintaining redox homeostasis in the bone microenvironment, thereby supporting bone regeneration. Mechanistically, antioxidant peptides can neutralize free radicals through electron or hydrogen donation, chelate pro-oxidant metal ions, inhibit lipid peroxidation, and activate endogenous antioxidant defense systems. Several studies indicate that these peptides can enhance the activity of antioxidant enzymes, including superoxide dismutase, catalase, and glutathione peroxidase, or activate redox-sensitive pathways, such as nuclear factor erythroid-2-related factor 2 (Nrf2) signaling [81].

Bone molecular metabolism is regulated by multiple signaling pathways. Key regulators include bone morphogenetic protein bone morphogenetic proteins/SMAD proteins (BMP-SMAD) and mitogen-activated protein kinase (MAPK) pathways [82]. These pathways translate extracellular stimuli into transcriptional events that control osteogenic differentiation [[83], [84], [85], [86], [87]]. In general, MAPK signaling involves sequential phosphorylation of upstream kinases, culminating in the activation of extracellular signal-regulated kinase (ERK), C-Jun N-terminal kinase (JNK) and p38. These kinases regulate transcription factors linked to cell differentiation and extracellular matrix production. Similarly, BMP signaling is initiated by BMP binding to type I and type II serine/threonine kinase receptors, leading to phosphorylation of SMAD1/5/8. Activated SMADS form complexes with SMAD4 and regulate osteogenic gene expression in the nucleus.

**Fig. 5:**
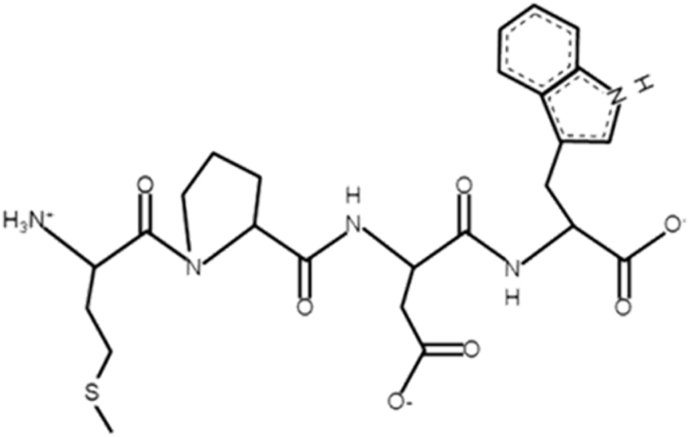
Chemical structure of microalgae therapeutic peptide with osteoblast differentiation activity. Peptide structure was generated using PepDraw [77].

**Fig. 6:**
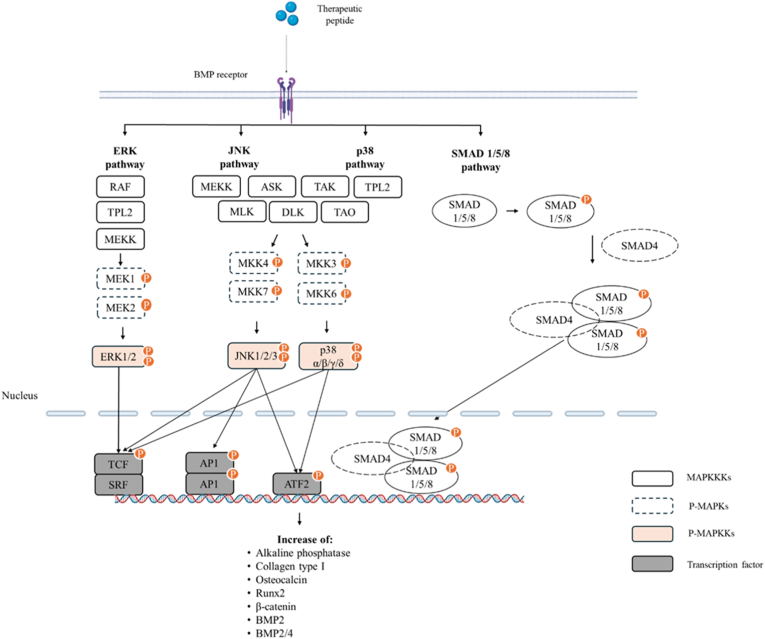
Hypothesized mechanism of bone regeneration triggered by microalgae therapeutic peptides, based on findings of Nguyen et al. (2013) [16] and Moradi et al. (2024) [17].

Despite this knowledge, the mechanisms through which microalgae therapeutic peptides promote bone regeneration are not fully elucidated. Currently, a detailed pathway analysis has been reported in a single study, using the tetrapeptide MDPW (Fig. 5) isolated from *Nannochloropsis oculata*. This therapeutic peptide has demonstrated the potential to promote osteoblast activity and modulate signaling pathways involved in bone formation (Fig. 6).

In the study by Nguyen et al. (2013) [16], it was evaluated the therapeutic peptide in human osteoblast-like MG-63 cell line and in murine mesenchymal stem cells (D1 cells). The tetrapeptide MDPW exposure resulted in a significant increase in BMP-2 gene and protein expression in both tested cell types. This observation suggests that BMP signaling may be involved in the osteogenic response. However, direct activation of BMP receptors or peptide-receptor interactions were not investigated. Downstream signaling analysis showed increased phosphorylation of SMAD1/5/8 following peptide treatment in MG-63 and D1 cells. These phosphorylation events were detected by Western Blott and are consistent with activation of the canonical BMP-SMAD pathway. Nevertheless, receptor-level association and SMAD nuclear translocation were not assessed, leaving the upstream mechanism unknown. The same study also demonstrated activation of mitogen-activated protein kinases. Specifically, phosphorylation of ERK1/2, JNK and p38 were high in both cell models. In MG-63 cells, MAPK phosphorylation increased progressively with peptide concentration. In contrast, D1 cells showed maximum ERK and p38 phosphorylation at intermediate concentrations. Followed by a partial decline at higher doses. Increased phosphorylation of SMAD1/5/8 and MAPKs was associated with high alkaline phosphatase (ALP) activity and enhanced expression of osteogenic markers, including collagen type 1 and osteocalcin. These changes were observed in both osteoblastic and mesenchymal stem cells, supporting the role of the microalgae therapeutic peptide in promoting osteogenic differentiation *in vitro*. Additional evidence from a study using a mix of peptides from *Arthrospira platensis* reported increased expression of RUNX2, osteocalcin, and β-catenin, along with higher ALP activity in human amniotic mesenchymal stem cells [17].

Overall, available data indicates that the identified microalgae therapeutic peptides can stimulate osteogenic differentiation and are associated with phosphorylation of ERK, JNK, p38 and SMAD1/5/8 in cell-based models. Nonetheless, *in vitro* and *in vivo* mechanistic knowledge in this specific niche is lacking. Moreover, the identification of upstream receptors, the pathway initiation, and the causal relationships between signaling cascades have not been established. Hence, addressing these gaps will be essential to validate these peptides as therapeutics for bone regeneration.

## 5 Advanced delivery systems for microalgae therapeutic peptides

**Fig. 7:**
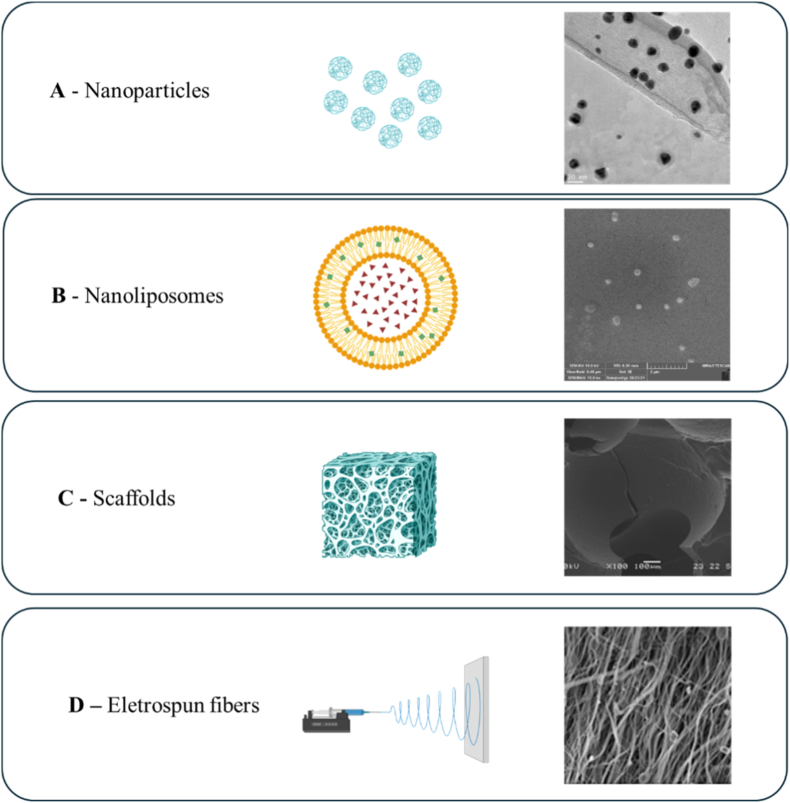
Microalgae therapeutic peptides delivery platforms reported in the literature: **A -** TEM images of peptide-functionalized gold nanoparticles [88]; **B -** nanoliposome with *Spirulina* hydrolysate [89]; **C –** incorporation of microalgae therapeutic peptide and catalyzed silica particle into PU foam [90]; **D –**polycaprolactone and *k*-carrageenan nanofibers with a microalgae therapeutic peptides concentration of 0.6% [91].

Despite limited application into advanced delivery systems for bone tissue engineering, microalgae therapeutic peptides have been effectively incorporated into engineered biomaterials. Microalgae therapeutic peptides can be integrated into diverse delivery platforms, including nanoparticles, nanoliposomes, foam-based scaffolds, and electrospun fibers (Fig. 7). These platforms aim to enhance peptide stability, bioavailability, and controlled release, thereby maximizing their therapeutic potential [92,93].

For instance, microalgae therapeutic peptides have been integrated into nanoparticle-based delivery platforms to enhance their stability, bioavailability, and controlled release. Torres-Dias et al. (2022) [88] incorporated a peptide derived from *Chlorella* sp. (VECYGPNRPQF) into gold nanoparticles (Fig. 7A), through surface functionalization. These exhibited increased antioxidant capacity and decreased cytotoxicity in *Aliivibrio fischeri*, a bacterium that is a model microorganism to monitor the toxicity and bioavailability of the microalgae therapeutic peptide gold nanoparticle [88]. Similarly, a protein hydrolysate from *Spirulina* sp. was encapsulated within nanoliposomes (Fig. 7B) to enhance wound healing in human foreskin fibroblast (HFFF-2) cell line. Human Foreskin Fibroblast (HFFF-2) cell line. *In vivo* assays performed in mice showed accelerated wound healing process through the increasing of the expression of bFGF, CD31 and COL1A [89]. Although this study was focused on skin regeneration, this approach highlights the versality of microalgae therapeutic peptides when combined with nano carriers, an approach translatable to bone regenerative therapies.

Microalgae therapeutic peptides can also be incorporated into porous foam-based scaffolds (Fig. 7C). Min et al. (2024) [90] reported the incorporation of a peptide derived from the marine microalgae *Thalassiosira oceanica* into polyurethane (PU) foam. This peptide was chemically modified by N-terminus amidation and C-terminus acetylation, while preserving the core sequence in the middle, to enhance its stability. This engineered peptide exhibited dual functionality, promoting silica deposition and antimicrobial activity against *E. coli* and *S. aureus*. Additionally, the incorporation of the peptide into the PU foam resulted in increased water holding capacity and mechanical properties. The ability of the peptide to induce silica deposition is of particular interest for bone tissue engineering, as silica has been shown to stimulate osteoblast differentiation and enhance bone mineralization *in vitro* [94].

Electrospinning has also been used to create peptide-based nanofibrous structures [95,96]. Raghunathan et al. (2024) [91], integrated low molecular with microalgae therapeutic peptides (<10 kDa) into polycaprolactone and *k*-carrageenan matrices to produce electrospun nanofibers (Fig. 7D). This biocomposite presented enhanced antimicrobial activity against *Escherichia coli* and *Staphylococcus aureus*. Additionally, this biocomposite demonstrated compatibility with human embryonic kidney (HEK) cell line. In another study, an antioxidant peptide (MLRSIGIPARL) discovered in *Arthrospira platensis* was integrated into electrospun chitosan/polyvinyl alcohol [97]. The integration of the peptide into the electrospun fibers maintained both bioactivity and biocompatibility, as demonstrated using mouse embryonic fibroblast (NIH-3T3) cell line. Although these materials were first designed for wound healing applications, it highlights the potential of microalgae therapeutic peptide nanofibrous structures as a possibility for bone tissue engineering applications, particularly when antimicrobial and antioxidant activities are needed.

3D printing has emerged as a suitable manufacturing technology, particularly in the development of scaffolds for tissue regeneration that may be personalized to each patient. However, to the best of the authors’ knowledge, there are currently no reports describing the use of microalgae therapeutic peptides in 3D printing for tissue engineering applications. Despite this gap, hydrogel-based systems represent a promising future direction for integrating microalgae therapeutic peptides into 3D printing.

**Fig. 8:**
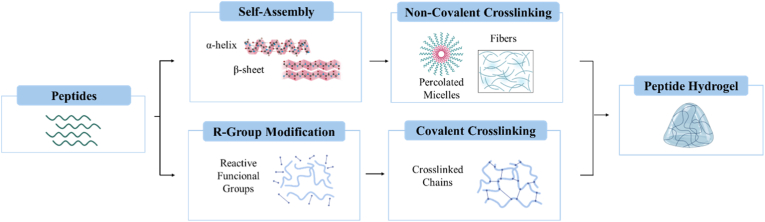
Peptide hydrogel fabrication through non-covalent and covalent strategies.

Peptide-based hydrogels can act either as injectable delivery systems or as printable hydrogels for extrusion-based 3D printing [98]. In extrusion-based printing, hydrogels are deposited layer-by-layer to form a scaffold. To do this, the hydrogel must have adequate rheological properties that enable both smooth extrusion and shape fidelity [99] and also peptide stability and biological activity must be preserved under shear stress during printing. These macroscopic properties (i.e. viscosity, stiffness) are strongly influenced by peptide sequence. Peptide-based hydrogels can be engineered through self-assembly [100] or covalent crosslinking combinations (Fig. 8) to fine-tune mechanical properties, gelation kinetics, and print fidelity.

For instance, the presence of aromatic or bulky hydrophobic residues enhances π–π and hydrophobic interactions. This result in denser fiber networks and higher mechanical properties, which improves printing fidelity. In contrast, incorporating charged or hydrophilic residues reduces viscosity and promotes shear thinning. However, excessive presence of these residues may reduce mechanical stiffness. This molecular tunability enables peptide-based hydrogels to meet the rheological requirements of extrusion-based printing [101].

Self-assembly hydrogels typically form through non-covalent interactions such as hydrogen bonding, π–π stacking, and hydrophobic interactions. This self-assembly creates secondary structures, including α-helices, β-sheet and hairpin motifs. These secondary structures not only determine the hydrogel nanofibrous morphology but also influence its mechanical strength and cellular interactions [102]. As a result, peptide hydrogels have high water content and tunable mechanical characteristics, both of which are essential to maintain cell viability and support tissue regeneration [102]. Alternatively, covalent strategies introduce permanent crosslinks to reinforce mechanical stability. Functionalization with reactive functional groups (i.e. methacrylate, acrylate, thiol) enables photo-crosslinking, enzymatic catalysis or click-chemistry approaches that produce structurally reinforced networks [103].

Beyond mechanical performance, biocompatibility is also a critical consideration. An additional advantage of peptide-based hydrogels is their capacity for functionalization. Bioactive sequences can be incorporated, providing flexible control over cellular signaling. For example, osteogenic functional motifs, such as RGD sequence, which enhances cell adhesion through integrin binding, can be efficiently included during synthesis.

## 6 Microalgae therapeutic peptides: from bench to clinic

The clinical translation of microalgae therapeutic peptides involves a structured roadmap from laboratory discovery to therapeutic application. Early-stage research focus on peptide identification, design, and functional characterization. During pre-clinical development, promising peptides are screened regarding their biological activity, stability, and preliminary safety *in vitro* assays are performed. As development progresses towards scale-up, good manufacturing practices become critical. Large-scale microalgae cultivation and extraction methods should be standardized to ensure batch-to-batch consistency, purity, and safety. This includes controlled microalgae growth conditions, validated extraction and purification processes, and quality assurance systems. Promising therapeutic peptides are subsequently evaluated in cell cultures and animal models to assess pharmacokinetics, pharmacodynamics and safety. Formulation optimization is often required to improve peptide stability, delivery, and bioavailability. Clinical trials then systematically evaluate safety and efficacy in humans. These studies must comply with good clinical practices to ensure ethical trial conduct, patient protection, and reliable data. Thus, at each stage of this translational pipeline, regulatory considerations increasingly shape experimental design, manufacturing strategies and validation requirements.

Accordingly, regulatory oversight by agencies, such as Food and Drug Administration (FDA), European Medicines Agency (EMA), and the International Council for Harmonization (ICH) are necessary to consider for the production and peptides application as therapeutic agents. As the number of peptides advancing through the pipelines of biopharmaceutical companies continues to increase, it becomes essential to standardize characterization tools to establish product specifications. These standards ensure the quality, effectiveness, and safety of these biological products at every stage of production, from obtaining raw feedstock to confirm the stability of the target therapeutic peptide.

The Center for Drug Evaluation and Research (CDER) and the Center for Biologics Evaluation and Research (CBER) at the US FDA, along with the EMA, established the standards to guarantee the safety, effectiveness, and quality of biological products [104] (Table 4). The ICH Q6B [105] recommendations are an essential source to define therapeutic peptides biological activity and purity. This guideline aims to set standards for tests such as visual inspection, biological activity assays, immunochemical evaluation, purity testing, and quantification techniques. In addition, ICH S6 (R1) provides a framework for the non-clinical safety evaluation of biotechnology-derived products, including biologically derived peptides, with particular emphasis on immunogenicity assessment [106]. ICH S8 complements this guidance by addressing the evaluation of immunotoxic effects, thereby supporting a comprehensive assessment of immune system safety [107]. Furthermore, ICH Q5E is relevant for biologically derived peptides as it outlines principles for demonstrating comparability according to manufacturing processes, ensuring product quality, safety, and efficacy [108]. ICH Q1A (R2) [109] standards provide guidance for the stability assessment of biological products, being crucial to determine shelf life, storage, and analyzing potential degradation pathways.

**Table 4:** Evaluation criteria used for the analysis of biologics and validation of bioanalytical procedures according to ICH guidelines.

	Regulatory Guideline	Evaluation Criteria
Analysis of Biologics	ICH Q6B	• Visual inspection • Biological activity assays • Immunochemical evaluation • Purity testing • Quantification procedures • Chemical changes • Structural and conformational changes • Tendency for aggregation • Activity assay
	ICH Q1A (R2)	• Stability studies
	ICH Q5E	• Comparability of biotechnological/biological products
	ICH S8	• Immunotoxicity studies for human pharmaceuticals
	ICH S6 (R1)	• Immunogenicity evaluation
Validation of Analytical Procedures	ICH Q14 ICH M10 ICH Q2 (R2)	• Selectivity/specificity • Range • Accuracy • Precision • Carryover • Dilution integrity • Robustness • Stability • Reinjection reproducibility

Validating analytical peptide characterization involves the implementation of specific methodologies and precautions. As a result, validation parameters for substances intended for use as pharmaceuticals have been updated, and relevant bioanalytical procedures have been incorporated into ICH M10 [110]. The most recent updates to the quality assurance standards for the validation of analytical procedures are documented in ICH Q14 [111] and included in ICH Q2 (R2) [112] and ICH M10 [110]. These updates ensure that the entire method validation process and its use in biopharmaceutical quality control reflect the most recent state of the art and meet modern standards.

Regulatory compliance is critical for safe and clinical translation of microalgae therapeutic peptides. Even though microalgae peptides are considered GRAS for food and feed applications, this cannot be directly translatable into therapeutic agents for bone regeneration. These must require preclinical and clinical validation. Although ICH guidelines provide general frameworks for biologically derived peptides, microalgae therapeutic peptides present product-specific regulatory challenges. Microalgal biomass may contain contaminants, such as heavy metals or microbial residues. However, these risks can be mitigated through regular testing, quality control and purification strategies. Compared with synthetic peptides, which are chemically defined and reproducible, microalgae therapeutic peptides are biologically derived, and may vary between batches, requiring extensive characterization, safety testing and good manufacturing compliant production.

Hence, by following a phased roadmap and addressing product-specific regulatory challenges, microalgae therapeutic peptides have the potential to go from experimental bioresources to clinically approved therapies in regenerative medicine. This translational potential is reflected in the growing number of patents involving microalgal therapeutic peptides for biomedical applications [[113], [114], [115], [116], [117]]. For instance, researchers have patented a food supplement with peptides as the active ingredient obtained from the microalgae *Navicula incerta*. This supplement protects the patients' liver cells and inhibits liver fibrosis [118]. A peptide isolated from the marine microalgae *Pavlova lutheri* contributed to the patenting of a pharmaceutical formulation for preventing and treating cancer [119]*.* The species *Nannochloropsis oculata* was used to extract a peptide (MPDW) through enzymatic hydrolysis and to develop a pharmaceutical drug for the prevention or treatment of bone diseases was also patented [114]. Another patent addressed the use of microalgae therapeutic peptides to stimulate osteoblast differentiation through the mitogen-activated protein kinase (MAPK) and nuclear factor kappa-light-chain-enhancer of activated B cells (NF-kB) pathways. The discovered therapeutic peptide has a molecular weight less than or equal to 10,000 Da, and its biological activity is easily maintained. It has high biological activity, is quickly absorbed, and its application is safe in the human body [113].

To this date, there is only one clinical trial involving microalgal therapeutic peptides on regenerative medicine, highlighting a starting shift from research and development towards clinical application. For instance, *Spirulina* therapeutic peptide is currently a useful adjuvant therapy in periodontal surgery that improves overall periodontal health and patient recovery. It specifically addresses the use of an algal peptide in a clinical environment for a particular regenerative (wound healing) purpose, even though this is an early-phase trial and more research is needed to validate and generalize the results [120].

## 7 Conclusions

Microalgae offer a sustainable source of therapeutic peptides that are particularly promising for bone regeneration. Reported microalgae therapeutic peptides exhibit anti-inflammatory, antibacterial, antioxidant and osteogenic properties, all of which are essential to enhance implant integration and bone repair. The multifunctionality of these peptides support their prospective integration into advanced biomaterials and medical devices aiming for bone regeneration. For instance, peptide-based hydrogels provide biomimetic and highly customizable platforms, contributing to precision medicine. Their integration with additive manufacturing techniques (e.g. 3D printing) enables the production of patient-specific implants with controlled architecture. However, translating these systems into clinical practices depends on the establishment of standardized workflows, including microalgae cultivation, therapeutic peptide extraction, purification, characterization, and formulation. Each process must be carefully controlled to ensure batch-to-batch consistency and reproducible therapeutic activity. In this context, adherence to international regulatory frameworks, such as those established by ICH, together with advanced analytical tools and validation protocols, is essential to ensure product safety, efficacy, and quality.

Currently, persistent bottlenecks, including limited clinical evidence, potential immunogenicity, variability among microalgae species, and the cost and complexity of large-scale microalgae therapeutic peptide production, must be systematically addressed. Therefore, future progress in this field of research will require a systematic and translational research strategy. In the short term, emphasis should be placed on standardized extraction and hydrolysis protocols, as well as robust *in vitro* and *in vivo* studies to validate therapeutic activity and safety. In parallel, there is a clear need for mechanistic research to elucidate the molecular pathways through which microalgae therapeutic peptides influence osteogenesis. In the medium time frame, focus should converge toward the development of functional scaffold prototypes and the initiation of early-phase clinical studies targeting bone regeneration.

In conclusion, despite existing scientific and translational challenges, microalgae therapeutic peptides represent a promising yet largely unexploited resource for bone regeneration. Considering that only a small portion of the vast microalgal biodiversity has been explored for bone regeneration, there are numerous prospects for the discovery of novel therapeutic peptides.

## CRediT authorship contribution statement

**Diana Pacheco:** Conceptualization, Formal analysis, Funding acquisition, Writing – original draft, Writing – review & editing. **Tatiana M.F. Patrício:** Conceptualization, Formal analysis, Funding acquisition, Supervision, Writing – review & editing. **Abílio J.F.N. Sobral:** Conceptualization, Formal analysis, Funding acquisition, Supervision, Writing – review & editing. **Telma Encarnação:** Conceptualization, Formal analysis, Funding acquisition, Supervision, Writing – review & editing.

## Declaration of competing interest

The authors declare that they have no known competing financial interests or personal relationships that could have appeared to influence the work reported in this paper.

## Data Availability

No data was used for the research described in the article.
